# Orthopoxvirus Seroprevalence in Cats and Veterinary Personnel in North-Eastern Italy in 2011

**DOI:** 10.3390/v11020101

**Published:** 2019-01-25

**Authors:** Daniele Lapa, Anna Beltrame, Alessandra Arzese, Fabrizio Carletti, Antonino Di Caro, Giuseppe Ippolito, Maria Rosaria Capobianchi, Concetta Castilletti

**Affiliations:** 1 National Institute for Infectious Diseases Lazzaro Spallanzani, 00149 Rome, Italy; daniele.lapa@inmi.it (D.L.); fabrizio.carletti@inmi.it (F.C.); antonino.dicaro@inmi.it (A.D.C.); giuseppe.ippolito@inmi.it (G.I.); maria.capobianchi@inmi.it (M.R.C.); 2 Centre for Tropical Diseases, IRCCS Sacro Cuore Don Calabria Hospital, 37024 Negrar VR, Italy; anna.beltrame@sacrocuore.it; 3 Medical Department (DAME), University of Udine, 33100 Udine, Italy; Alessandra.arzese@asuiud.sanita.fvg.it; 4 Microbiology Laboratory Unit, Azienda Sanitaria Universitaria Integrata, Santa Maria della Misericordia University Hospital, 33100 Udine, Italy

**Keywords:** Orthopoxvirus, seroprevalence, cat, veterinarian, Italy

## Abstract

Orthopoxviruses (OPV) are emerging zoonotic pathogens, and an increasing number of human infections is currently reported in Europe and in other continents, warranting heightened attention on this topic. Following two OPV infections reported in veterinarians scratched by sick cats in 2005 and 2007 in North-Eastern-Italy, involving a previously undescribed OPV, a similar strain was isolated by a sick cat from the same territory in 2011, i.e., 6 years later, raising attention on OPV circulation in this region. A surveillance program was launched to assess the OPV seroprevalence among the veterinarians working in local veterinary clinics and in the local wild and domestic cat population; seroprevalence was 33.3% in veterinarians and 19.5% in cats. Seroprevalence in cats was unevenly distributed, peaking at 40% in the area where OPV-infected cats had been observed.

## 1. Introduction

*Orthopoxvirus* (OPV) is a genus of the family *Poxviridae*, including multiple species isolated from mammals, most of which are zoonotic viruses threatening human health. While naturally occurring smallpox infections are indeed no longer a reason of concern (except in a bioterrorist context), after the end of routine smallpox vaccination in 1977, an increased number of human infections with zoonotic OPV such as Monkeypox virus or Cowpox virus (CPXV) [1,2,3,4,5] occurred, mainly affecting young people [6]. Reports of OPV infections in animals and humans have largely increased during recent decades, which has enhanced the perception of zoonotic potential of OPV and of increasing risk for humans.

CPXV is the only known wildlife-borne OPV in Europe [7,8]; it is a zoonotic agent and is considered the most common one [1]. Various animal species as well as humans are susceptible to the infection [9,10,11]. Cases of transmission from pet rats to humans have been reported in northern France [12] and in Germany [13,14,15,16] and these animals are emerging as a novel potential source of local outbreaks of OPV zoonoses [17]. Small rodents are believed to be the current reservoir of CPXV [18], whereas cats and cows are the most relevant source of transmission to humans [19] from 0.3% (Turkey) up to 42.4% (Germany) [7,20,21,22]. Since the first report about a CPXV-infected domestic cat in Great Britain in 1977 [23], further cases have been documented in Europe [24,25]. Cats play a link between the natural reservoirs and humans in the urban environment [1,25,26]. To date, at least five monophyletic clades of CPXV have been identified, fulfilling the requirements to be considered as distinct viral species [27,28]

Moreover, in both Great Britain and Germany CPXV infection of cats has been recognized as a seasonal disease with a peak incidence in autumn [29]. In cats, the disease originates from exposure to infected rodents; the clinical course varies from no symptoms to widespread skin necrotic lesions and can ultimately lead to death [19]. Cutaneous clinical signs in infected cats include ulcerated, erythematous and crusted lesions, predominantly on the head, neck and forelegs.

Despite the increased attention to this topic, there are only few studies on OPV seroprevalence in European cats, ranging from 2% to 13.0% [24,30,31,32,33]; however, OPV seroprevalence in Italian cats has not been addressed to date.

In the last decade, only three cases of OPV zoonotic cases were described in Italy, two of them occurring in veterinary personnel scratched by sick domestic cats in 2005 and 2007. Although only partial characterization of these OPV isolates was obtained, preliminary data supported that these strains did not belong to known OPV species, and also indicated that OPV were circulating in local fauna [25]. The third OPV zoonotic case was observed in the Lazio region during an OPV outbreak that occurred in January 2015 in a colony of *Macaca tonkeana* (a species of primate in the family of *Cercopithecidae*) in the province of Rieti [34], which caused a human asymptomatic infection [2]. In this case, the extended genomic characterization of the isolate (OPV Abatino) showed that it may represent a novel clade of OPV, the nearest CPXV strain belonging to a novel CPXV lineage (CPXV-Ger2010-MKY) [27], closely related to, although distinct from, the *Ectromelia virus* species (ECTV). In addition, a virus very similar to the OPV Abatino has been recently isolated from a fatal feline infection in Tuscany [26]. These novel OPV isolates, that may be the result of complex evolution, indicate that previously reported cases in Italy could represent the tip of an iceberg yet to be explored [35].

Taken together, the results of OPV surveillance in Italy show that the circulation of strains with zoonotic potential may result from both local segregation and genetic evolution, and importation of strains from other countries, leading to obsolescence the concept that the Alpes barrier historically defended the Italian territories from OPV spread.

This paper describes the results of a surveillance program that was launched in 2011 among local veterinary clinics of Friuli Venezia Giulia (FVG) to establish the OPV seroprevalence in veterinarians and cats, as an indirect indicator of the extent of the circulation of these viruses in the human and feline local populations.

## 2. Materials and Methods

### 2.1. Sample Collection

From May 2010 to October 2011, a total of 36 veterinarians and 226 cats (>1 year old), selected from 11 veterinary clinics located in nine different areas of FVG territory, were included in the study. Human and animal blood samples were sent to the Laboratory of Virology at the National Institute for Infectious Disease “L. Spallanzani” in Rome, where serological investigation was performed.

A standardized questionnaire was used to record the past history, as well as to evaluate possible risk factors of veterinaries and cats. Exposure rate of veterinarians was measured on the basis of years of work experience and weekly exposure to cats. Individual written consent was obtained from all participant veterinarians before applying the survey and drawing the blood samples. Individual written consent was also obtained from the cat owners.

### 2.2. Challenge Virus Stock Preparation

A Vaccinia virus (VACV, Lancy-Vaxina strain, Berna Biotech Ltd, Berna, Swiss) was propagated in Vero E6 cells and harvested when 70% to 80% of the cell monolayer showed cytopathic effect (CPE). After three cycles of freezing-thawing, cell lysates were clarified by low-speed centrifugation, aliquoted and stored at –70 °C. The VACV virus stock used for all the experiments titered 10^7.8^ plaque forming units (PFU)/mL.

### 2.3. Serologic Tests

Serum samples from both cats and veterinarians were stored at –20 °C until use. The presence of OPV-specific antibodies was assessed by immunofluorescence (IFA) and confirmed by microneutralization (MNA) assay.

Slides for IFA were prepared in the laboratory, using Vero E6 cells infected with VACV. Specific human IgG were detected using standard procedures [36]. In particular, rabbit anti-human IgG conjugated with fluorescein isothiocyanate was used as a secondary antibody (Sigma-Aldrich, Inc.). Negative and high-positive controls were included in each test. An IgG IFA titer ≥1:40 was scored as positive.

MNA was performed according to a previously published method [37,38], using VACV for the challenge. A serum from a previously (4 years) vaccinated subject was used as positive control. An MNA titer ≥1:20 was scored as positive. Human samples positive to both IFA and MNA were considered as true positive; cat serum samples were analyzed only by MNA.

### 2.4. Statistical Analysis

Chi square tests and Fisher exact tests were used as appropriate (Prism 5.0, Graphpad, California). A *p* ≤ 0.05 was considered significant.

## 3. Results

**Figure 1:**
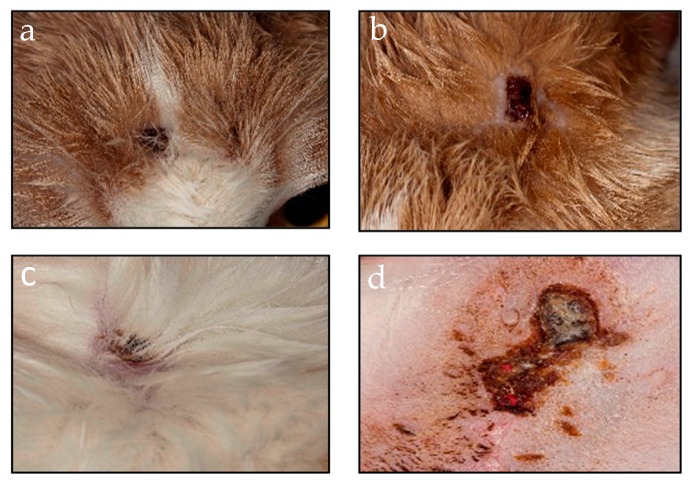
Clinical of the *Orthopoxvirus* (OPV)-infected cat. Skin lesions on the numerous vesicles spread on the muzzle (**a**), head (**b**), back (**c**) and in the mammary area (**d**) of the sick cat.

In February 2011, a sick cat with skin lesions suggestive of OPV infection was observed at a veterinary clinic located in Fagagna, FVG. The lesions, that had appeared one week before the visit, healed about 10 days after the visit (Figure 1); overall, the disease course appeared to be mild, and the cat recovered completely without sequelae.

The OPV diagnosis was based on OPV-specific PCR [25]; partial sequence characterization of the virus isolate, based on complete *hemagglutinin* (HA, 948 nt) and *crmB* (1050 nt) genes, indicated that this virus was similar to OPV isolates obtained from veterinarians exposed to sick cats 4 and 6 years before in the same geographical region [25], suggesting that OPV had been circulating in the region for years.

This prompted us to initiate a seroprevalence study in veterinarians and cats of the region, to establish the extent of OPV circulation and the risk factors for persons professionally exposed to wild and domestic cats.

The median age of the veterinarians was 41.5 years (range 25–57 years); 20 (55.6%) were female, 5 (13.8%) had underlying immunosuppression and 16 (44.4%) reported a previous smallpox vaccination. The median clinic work experience was 15.5 years (range, 1–31 years); among veterinarians, 24 subjects (66.7%) reported a weekly exposure to more than 10 cats and only nine (25%) subjects considered OPV infection a professional risk. Eleven (30.5%) veterinarians reported prior exposure to more than 10 cats presenting with ulcerative dermatitis.

The overall seroprevalence of OPV infection among veterinarians was 33.3% (12/36) with an increasing trend according to age (Table 1); no significant association with exposure rate was observed.

A total of 24 veterinarians resulted OPV-seronegative, including four who were vaccinated; all the remaining 12 seropositive veterinarians received previous smallpox vaccinations, but it is not possible to establish whether the OPV-seropositivity could result from boosting antibody responses after OPV exposure during the veterinary work.

**Figure 2:**
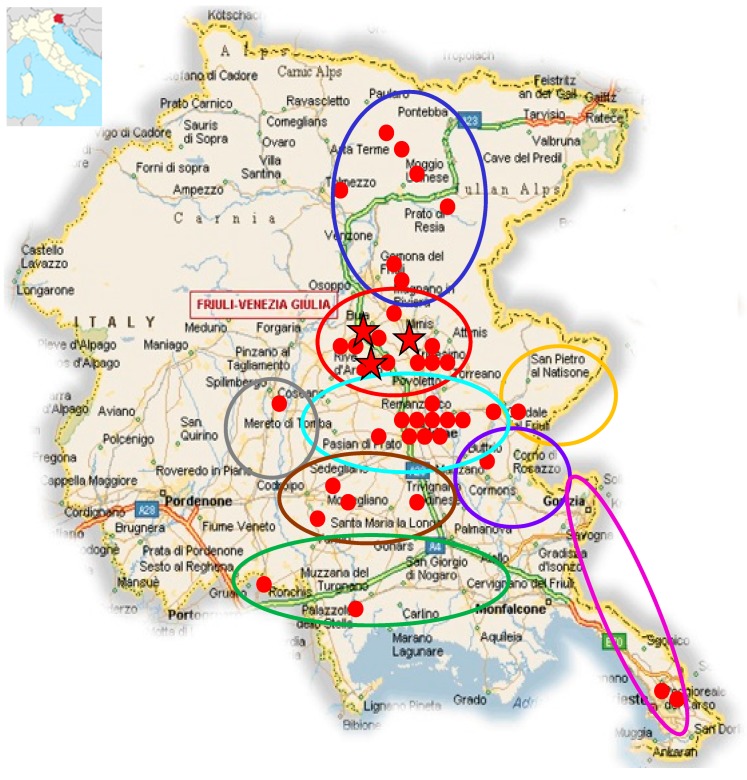
Geographic map of Friuli Venezia Giulia, Northeastern Italy, with the location of the nine areas of cat residence; red dots indicate seropositive cats, and red stars indicate the area in which sick cats were detected over 2005–2011. Municipalities included in the research areas were the following: (**A**) Bordano, Cavazzo carnico, Gemona, Moggio, Osoppo, Resia, Tolmezzo, Treppo Carnico, Venzone; (**B**) Buia, Cassacco, Colorendo di Montealbano, Fagagna, Majano, Ragogna, Rive d’Arcano, San Daniele, Tarcento, Treppo Grande, Tricesimo; (**C**) Basiliano, Campoformido, Feletto Umberto, Grions del Torre, Martignacco, Pagnacco, Pasian di Prato, Passons, Povoletto, Remanzacco, Tavagnacco, Udine; (**D**) Cividale, Premariacco, San Leonardo, San Pietro al Natisone; (**E**) Buttrio, Corno di Rosazzo, Manzano, Pavia di Udine, San Giovanni al Natisone; (**F**) Carso, Gorizia, Trieste; (**G**) Camino al Tagliamento, Castions di Strada, Codroipo, Galleriano, Mortegliano, Pozzuolo del Friuli, Rivignano, Talmassons; (**H**) Latisana, Muzzana, Palazzolo dello Stella, Portogruaro, Rivarotta di Teor, San Giorgio di Nogaro; (**I**) Casarsa, Spilimbergo.

In order to investigate the OPV seroprevalence in wild and domestic cats, 226 animals >1 year old, referred by 11 selected veterinary clinics of FVG were sampled from February 2010 to May 2011. The 226 cats analyzed had a median age of 6 (range 1–19 years); 134 cats (59.2%) were domestics and 108 (47.8%) lived in rural areas; 173 (76.5%) cats had been in contact with other cats while 163 (72.1%) with rats. The overall OPV seroprevalence among cats was 19.5% (44/226), with a great variability (ranging between 5% and 40%), depending on the area of residence (Table 2, Figure 2). The mean of MNA positive titers was 42.35 with no significant differences among the residence areas of the animals.

Seropositive cats were more frequent in the group of animals who had been in contact with other cats (21.9% of cats with contacts were seropositive vs. 9.4% of cats without contacts, *p* ≤ 0.05).

No significantly different seroprevalence was observed in cats living in urban environments vs. cats living in rural environments, as well as in cats exposed or not exposed to rats. The seroprevalence trend according to cat age was not significant.

## 4. Discussion

OPV with zoonotic potential are persistently circulating in FVG, and may represent a relevant professional risk for persons exposed to animals. We launched a large seroepidemiological study, to establish the OPV seroprevalence in cats and in veterinary clinic personnel from different areas of the FVG region.

The overall seroprevalence in cats was 19.5%, and was higher than the prevalence observed in previous studies in Europe (2% to 13.0%) [24,30,31,32]. Interestingly, a high geographic heterogeneity was observed within the study region, with seroprevalence ranging from 5% and 40%; the highest frequency was observed in the area where sick cats had been observed in the previous [25] and in the present study. The only identified cat risk factor was having had contact with other cats.

The overall seroprevalence in veterinarians was 33.3%, with an increasing trend according to age, while no significant association with exposure rate was observed. All veterinarians showing OPV antibodies had received previous smallpox vaccinations (notably, four of the vaccinated persons did not show detectable OPV antibodies), while none of the non-vaccinated veterinarians showed positive OPV specific antibodies. Hence, increased age and previous smallpox vaccination appear to be the main factors associated with the presence of OPV-specific antibodies. In other words, these findings do not allow us to establish whether the antibody titer observed in the veterinarians represent an anamnestic response to original vaccination favored by repeated work-related exposure, or merely reflect humoral immune memory to vaccination.

The observations of this study might be alarming because OPV vaccination has been globally discontinued since the late 1970s. This has resulted in reduction of protective immunity, not only against smallpox virus, but also against a variety of other OPV over time, raising the chance of new OPV cases in humans. OPV infection, although relatively rare in animals and uncommon in humans, has gained some recognition through micro-outbreaks in recent years, and raised attention also due to recently imported human cases of Monkeypox infections [3,4,5]. Moreover, OPV infection of humans is not a notifiable disease in Italy, virological diagnosis relies on specialized laboratories, and is often not included in the differential diagnosis. Increased public awareness and linkage between human and veterinary health authorities is necessary to improve public health measures in the control of zoonotic OPV.

## Figures and Tables

**Table 1:** OPV-specific antibodies in veterinarians, Friuli Venezia Giulia (Northeastern Italy).

Veterinarians (Years)	n	Positive	Prevalence (%) *
≤30	5	0	0
31–40	13	2	15.4
41–50	10	5	50
>50	8	5	62.5

* *p* = 0.0078, chi-squared test for trend.

**Table 2:** Seroprevalence of animals according to the residence area.

Area of Residence	Positive/Analyzed Cats	Seroprevalence
A (blue)	7/19	36.8%
B (red)	10/25	40%
C (turquoise)	11/72	15.3%
D (yellow)	1/8	12.5%
E (purple)	1/10	10%
F (pink)	2/40	5%
G (brown)	4/22	18.2%
H (green)	2/7	28.6%
I (grey)	1/3	33.3%
Not known	5/20	25%
Overall	44/226	19.5%
